# Role of Cajal Bodies and Nucleolus in the Maturation of the U1 snRNP in *Arabidopsis*

**DOI:** 10.1371/journal.pone.0003989

**Published:** 2008-12-22

**Authors:** Zdravko J. Lorković, Andrea Barta

**Affiliations:** Max F. Perutz Laboratories, Department of Medical Biochemistry, Medical University of Vienna, Vienna, Austria; University of Hong Kong, China

## Abstract

**Background:**

The biogenesis of spliceosomal snRNPs takes place in both the cytoplasm where Sm core proteins are added and snRNAs are modified at the 5′ and 3′ termini and in the nucleus where snRNP-specific proteins associate. U1 snRNP consists of U1 snRNA, seven Sm proteins and three snRNP-specific proteins, U1-70K, U1A, and U1C. It has been shown previously that after import to the nucleus U2 and U4/U6 snRNP-specific proteins first appear in Cajal bodies (CB) and then in splicing speckles. In addition, in cells grown under normal conditions U2, U4, U5, and U6 snRNAs/snRNPs are abundant in CBs. Therefore, it has been proposed that the final assembly of these spliceosomal snRNPs takes place in this nuclear compartment. In contrast, U1 snRNA in both animal and plant cells has rarely been found in this nuclear compartment.

**Methodology/Principal Findings:**

Here, we analysed the subnuclear distribution of *Arabidopsis* U1 snRNP-specific proteins fused to GFP or mRFP in transiently transformed *Arabidopsis* protoplasts. Irrespective of the tag used, U1-70K was exclusively found in the nucleus, whereas U1A and U1C were equally distributed between the nucleus and the cytoplasm. In the nucleus all three proteins localised to CBs and nucleoli although to different extent. Interestingly, we also found that the appearance of the three proteins in nuclear speckles differ significantly. U1-70K was mostly found in speckles whereas U1A and U1C in ∼90% of cells showed diffuse nucleoplasmic in combination with CBs and nucleolar localisation.

**Conclusions/Significance:**

Our data indicate that CBs and nucleolus are involved in the maturation of U1 snRNP. Differences in nuclear accumulation and distribution between U1-70K and U1A and U1C proteins may indicate that either U1-70K or U1A and U1C associate with, or is/are involved, in other nuclear processes apart from pre-mRNA splicing.

## Introduction

Pre-mRNA splicing is mediated by the spliceosome, a dynamic macromolecular complex which assembles anew on each intron. Five small nuclear ribonucleoprotein particles (snRNPs) and hundreds of proteins take part in this process. Each snRNP is composed of one uridine-rich snRNAs and the seven common Sm (or Lsm, in the case of U6 snRNP) proteins, B/B′, D1, D2, D3, E, F and G. In addition to the Sm/Lsm proteins, each snRNP contains particle-specific proteins (see below). In the nucleus, the majority of the snRNPs localise in interchromatin granule clusters, also known as nuclear speckles, and in a diffuse nucleoplasmic pool [1], [2]. A smaller fraction of snRNAs, Sm proteins and some snRNP-specific proteins are also found in Cajal bodies (CB). In contrast, splicing factors that are not associated with snRNPs are excluded from these structures [2]–[5].

CBs are non-membrane nuclear bodies, of about 0.5–1.0 µm, which are present within the nuclei of most plant and animal cells. CBs are dynamic structures that move, split, rejoin and exchange their molecular contents with the surrounding nucleoplasm. The size and the number of CBs depend on cell type, cell cycle, and metabolic activity [6]–[13]. It is currently thought that CBs function in metabolism of different classes of RNP particles, e.g., spliceosomal snRNPs, small nucleolar RNPs, telomerase, and U7 snRNP. In addition, CBs were found in association with specific gene loci, such as histone and U2 snRNA gene clusters. Therefore, roles for CBs in regulation of gene expression and assembly and transport of macromolecular complexes have been proposed [3]–[5], [14]–[17].

SnRNP biogenesis is a stepwise process that starts in the nucleus, continues in the cytoplasm, and finishes in the nucleus. Newly synthesized snRNAs (except U6) are exported to the cytoplasm where core snRNPs are formed by the assembly of seven Sm proteins on each snRNA. This is followed by hypermethylation of the 5′ cap yielding 2,2,7-tri-methyl-guanosine (m_3_G). The m_3_G, together with Sm proteins, serves as a nuclear import signal. The SMN complex interacts with snurportin1, a snRNP import receptor, and facilitates nuclear import of snRNPs [17]–[19]. Binding of snRNP-specific proteins is required for the production of mature snRNPs that are active in splicing [20]. However, the cellular site(s) of this step in snRNP biogenesis and the timing with respect to other maturation steps are not well defined. Several U1- and U2-specific proteins are transported into the nucleus independently of their cognate snRNAs [21]–[25], implying that the final assembly occurs after import of the core snRNPs into the nucleus. A function for CBs in the biogenesis of snRNPs has been demonstrated by several recent studies. Upon import into the nucleus, newly formed snRNPs pass through CBs and nucleoli and only later accumulate in speckles [26]. Once back in the nucleus, snRNAs are modified by 2′-O-ribose methylation and pseudouridylation [14], [27]. These modifications are mediated by small RNAs that are localized in CBs (scaRNAs) [14], [27]–[30]. It has been shown that modification of U2 snRNA is required for the binding of several U2-specific proteins in *Xenopus* oocytes [31], [32]. Thus, it is conceivable that particle-specific proteins associate with the core snRNPs during their passage through the CBs. Indeed, several U2 snRNP-specific proteins, U2, U4, U5, and U6 snRNAs as well as the U4/U6 assembly factor SART3 have been detected in CBs either at the steady-state or after transient expression in human and plant cells [2], [33]–[38].

Among the five spliceosomal snRNP, the U1 snRNP is the simplest one. Besides the U1 snRNA and the common Sm proteins, the human (and most likely the plant) U1 snRNP contains three specific proteins: U1-70K, U1A, and U1C. U1-70K and U1A proteins bind directly to the U1 snRNA while the U1C protein is attached through protein–protein interactions with U1-70K and Sm proteins. U1-70K, U1A and U1C proteins are imported to the nucleus independently of the U1 snRNA [21], [22], [24], [25], [39], indicating that the final assembly of the U1 snRNP occurs in the nucleus. However, the role of CBs in this process is not clear. The U1 snRNA, in plant and animal cells, is not abundant in CBs [12], [33], [40] and so far, only one U1 snRNP-specific protein, the U1C protein, was detected in CBs [41].

In general very little is known about snRNP protein composition and biogenesis in plant cells [7], [8], [9], [33], [34], [42], [43]. In this work we set out to analyse the localisation of three *Arabidopsis* U1 snRNP-specific proteins in a transient expression system in *Arabidopsis* protoplasts. We could show that *Arabidopsis* U1 snRNP-specific proteins accumulate in CBs and nucleoli, indicating that the final assembly of U1 snRNP takes place in these two nuclear compartments.

## Results

### Expression and localisation of U1 snRNP-specific proteins in *Arabidopsis* protoplasts

**Figure 1:**
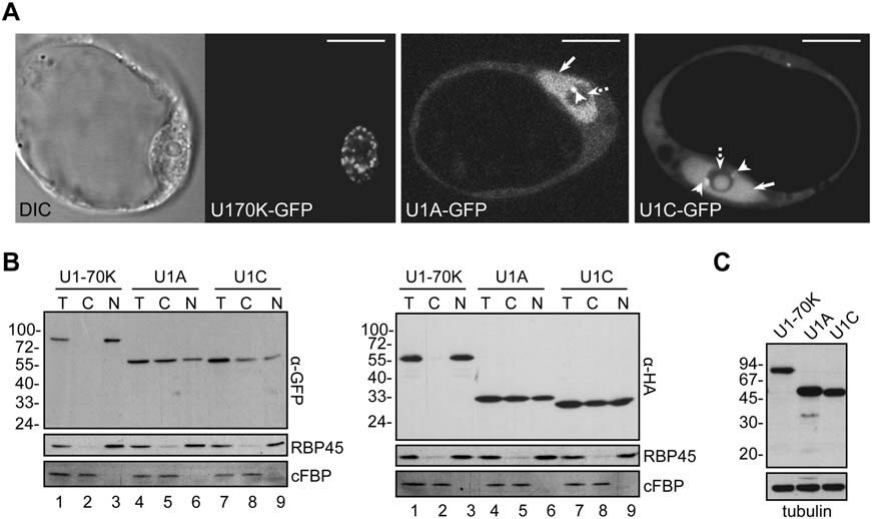
Localisation of transiently expressed U1 snRNP proteins in *Arabidopsis* protoplasts. (A) Single confocal sections of protoplasts expressing U1-70K, U1A, and U1C proteins fused to GFP. Corresponding differential interference contrast (DIC) image of a cell expressing U1-70K is also shown. Arrows, broken arrow and arrowheads point to nuclei, nucleoli and CBs, respectively. Scale bars, 15 µm. (B) Cellular localisation of GFP- (left panel) or HA-tagged (right panel) U1 snRNP-specific proteins studied by cellular fractionation. Cell extracts were fractionated as described [57]. Lanes T, C, and N; total cellular, cytoplasmic, and nuclear protein fractions, respectively. Proteins were resolved by SDS-PAGE and analyzed by Western blotting, using mouse anti-GFP and rat anti-HA mAb. Molecular mass standards in kDa are indicated on the left. To control the quality of the fractionation procedure the same blots were probed with antibodies against nuclear and cytoplasmic proteins RBP45 [58] and fructose 1,6-bisphosphatase (cFBP), respectively (two bottom panels). (C) Immunodetection of U1-70K, U1A and U1C GFP fusion proteins in protein extract from transformed protoplasts. Total protein extracts were analysed by SDS-PAGE and Western blotting with anti-GFP antibody. Molecular mass standards in kDa are indicated on the left. Western blotting of the same protein extracts with anti-tubulin antibodies was performed as a loading control (bottom panel).

We could previously show that transient expression of U2 snRNP-specific proteins in *Arabidopsis* protoplasts results in their correct localisation in the nucleus. In particular, it has been shown that U2B″ and U2A′ proteins localise in a diffuse/speckled nucleoplasmic pattern, with the majority of cells also showing localisation in CBs and to a lesser extent in nucleoli [34]. Therefore, we used this experimental system combined with confocal microscopy to analyse the localisation of *Arabidopsis* U1 snRNP- specific proteins, U1-70K, U1A and U1C. As can be seen from the Figure 1A all three proteins localised to the nucleus. However, U1-70K was exclusively found in the nucleus whereas U1A and U1C were found in the nucleus and in the cytoplasm in virtually all cells. As GFP is a rather large tag, it could possibly influence the import of small proteins (U1A and U1C) into the nucleus. Therefore, biochemical fractionation of protoplasts expressing GFP and HA-tagged proteins into nuclear and cytoplasmic fractions was performed. These experiments revealed that 24 hours after transformation approximately 50% of U1A and U1C proteins remained cytoplasmic irrespective of the tag used (Figure 1B, lanes 5 and 8). In contrast, U1-70K could not be detected in the cytoplasmic fraction (Figure 1B, lane 2). Analysis of the distribution of nuclear and cytoplasmic proteins RBP45 and fructose 1,6-bisphosphatase (cFBP) indicated that fractionation procedure was specific (Figure 1B, two bottom panels). We also compared the expression levels of proteins as this might influence nuclear import. However, analysis of GFP-tagged proteins revealed similar expression levels for all three proteins analysed (Figure 1C, upper panel; see also Figure 1B). Control western analysis with antibody against the tubulin revealed that equal amounts of proteins were loaded in each lane (Figure 1C, bottom panel). Finally, no major degradation products were observed for any of the three proteins (Figure 1B and 1C). Thus, the cytoplasmic localisation of U1A and U1C proteins is not due to their higher expression levels or protein degradation.

### Transiently expressed U1 snRNP-specific proteins are incorporated into mature snRNPs

**Figure 2:**
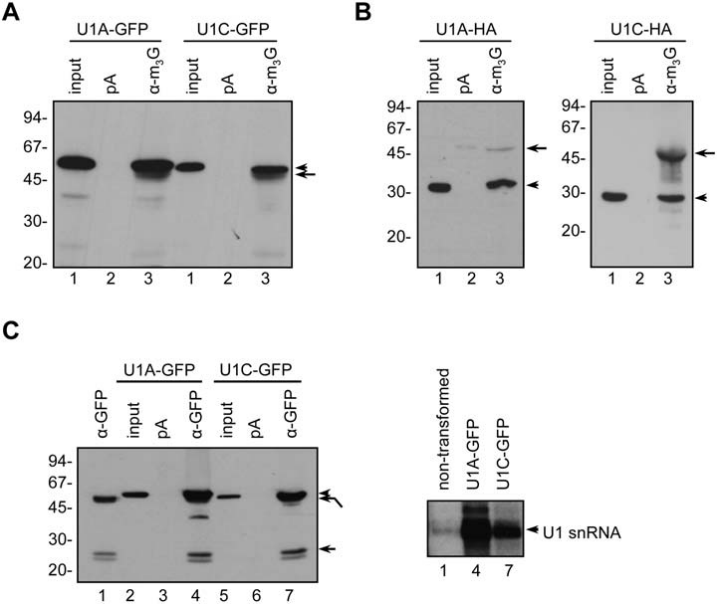
Transiently expressed U1 snRNP-specific proteins assemble into mature snRNP. (A) Immunoprecipitation of U1A-GFP and U1C-GFP fusion proteins with anti-m_3_G antibody (α-m_3_G). Lanes 1, input protein extract. Lanes 2, protein extracts incubated with protein-A Sepharose (pA). Lanes 3, immunoprecipitations with anti-m_3_G antibody (α-m_3_G). Arrowheads and arrows point to precipitated proteins and immunoglobulin heavy chains, respectively. The blot was probed with anti-GFP antibody. (B) Immunoprecipitation of U1A-HA and U1C-HA fusion proteins with anti-m_3_G antibody (α-m_3_G). The blots were probed with anti-HA antibody. Other details as in (A). (C) U1A-GFP and U1C-GFP fusion proteins precipitated with anti-GFP antibody co-immunoprecipitate U1 snRNAs. Left panel: lane 1, immunoprecipitation with anti-GFP antibody with protein extract from non-transformed protoplasts; lanes 2 and 5, input protein extract from cells expressing U1A-GFP and U1C-GFP fusion proteins, respectively; lanes 3 and 6, protein extracts from transformed cells incubated with protein-A Sepharose only (pA); lanes 4 and 7, immunoprecipitations with anti-GFP antibody (α-GFP) with protein extracts from transformed protoplasts. Arrowheads point to U1A and U1C GFP-tagged proteins and arrows point to immunoglobulin heavy and light chains. Right panel: analysis of anti-GFP immunoprecipitates (from the left panel, lanes 1, 4, and 7) for the presence of U1 snRNAs. After immunoprecipitation RNA was extracted, labelled by [^32^P]-pCp ligation and analyzed on 8% denaturing PAA gels. Lane 1, RNA immunoprecipitated with anti-GFP antibody from non-transformed cells. Lanes 4 and 7, RNA co-precipitated with U1A-GFP and U1C-GFP, respectively.

Transient expression of HA and GFP-tagged U2A′, U2B″ and U1-70K in *Arabidopsis* cell suspension protoplasts resulted in their correct assembly into mature snRNPs [34], [43]. To find out whether transiently expressed U1A and U1C proteins associate with U1 snRNP, immunoprecipitations with anti-m_3_G antibody which recognizes the trimethylguanosine Cap structure at the 5′ end of U snRNAs [44] were performed. Figure 2A and 2B demonstrates that GFP- and HA-tagged U1A and U1C proteins were efficiently precipitated with anti-m_3_G antibody (Figure 2A and 2B, lanes 3), indicating association with the mature snRNP. To further support this observation protein extracts form protoplasts expressing GFP-tagged proteins were immunoprecipitated with anti-GFP antibodies (Figure 2C, left panel) followed by RNA extraction. Analysis of immunoprecipitates for the presence of snRNAs by [^32^P]-pCp labelling revealed efficient co-precipitation of U1 snRNA with both proteins (Figure 2C, lanes 4 and 7 on the right panel), but not of other spliceosomal snRNAs. Immunoprecipitation performed with anti-GFP antibody and protein extracts from non-transformed cells indicated that the procedure was specific. Figure 2C (lane 1 in right panel) shows that no appreciable amounts of U1 snRNA were precipitated. From the immunoprecipitation data shown in Figure 2 and from our previous data [34], [43] we conclude that transient expression of U1 snRNP-specific proteins in *Arabidopsis* protoplasts results in efficient incorporation into mature U1 snRNPs.

### U1 snRNP-specific proteins accumulate in Cajal bodies and nucleoli

**Figure 3:**
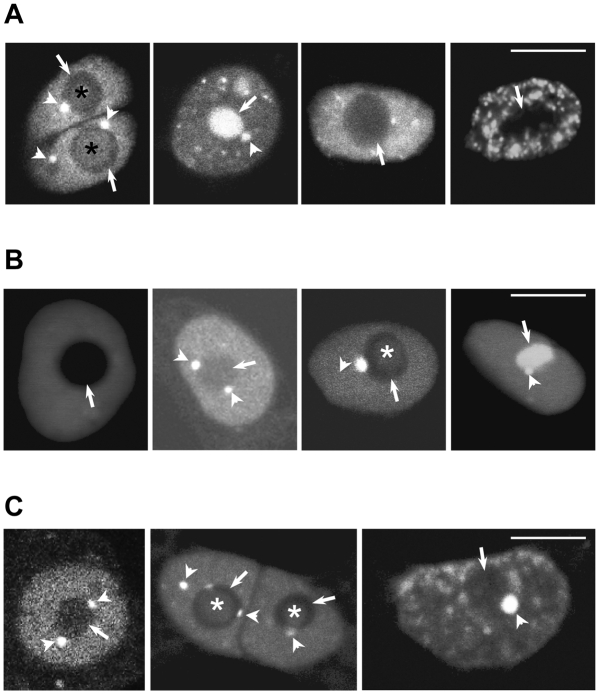
Nuclear distribution of transiently expressed U1-70K, U1A, and U1C proteins. Representative images of nuclear patterns observed in protoplasts expressing U1-70K (A), U1A (B), and U1C (C) GFP-tagged proteins. Single confocal sections are shown. Arrows, arrowheads, and asterisks point to nucleoli, CBs, and nucleolar cavities, respectively. Scale bars, 8 µm.

**Figure 4:**
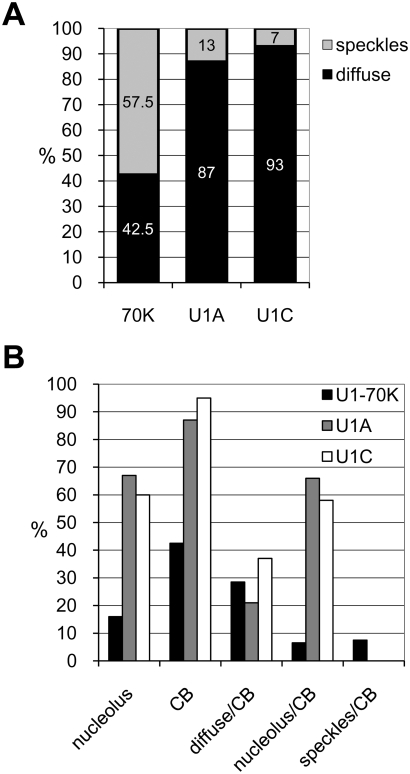
Quantification of nuclear patterns shown in Figure 3. Transformations with GFP tagged proteins were performed independently three times, and each time 100 randomly chosen cells were analysed. (A) Speckled and diffuse nucleoplasmic staining patterns were scored and percentages are indicated inside the bars. (B) CB and nucleolar localisation as well as combinations of different patterns as indicated on X-axis were scored. The Y-axis represents the percentage of cells showing specific pattern(s).

From the images shown in Figure 1A it is obvious that the three U1 snRNP-specific proteins show different localisation patterns in the nucleus. As we observed that the frequencies of the identified nuclear patterns differed between U1-70K and U1A/U1C proteins we performed quantitative analysis. To do so, three independent transformations of *Arabidopsis* protoplasts were performed and nuclear localisation patterns for the three U1 snRNP-specific proteins were scored by counting 100 cells for each protein. The most common patterns observed with U1-70K, U1A, and U1C proteins are shown in Figure 3A, 3B, and 3C, respectively. In general, we can say that all three proteins show: (i) diffuse nucleoplasmic staining with or without CBs localisation: (ii) diffuse nucleoplasmic staining with nucleolar localisation; (iii) diffuse nucleoplasmic staining with nucleolar and CBs localisation; (iv) speckled nucleoplasmic localisation with or without nucleolar and CBs staining. However, it is important to note that most cells showing speckled localisation did not show localisation in nucleoli and CBs and that in the majority of cells nucleolar staining was restricted to the nucleolar cavity. Next, we compared the percentages of cells showing speckled and diffuse nucleoplasmic localisation. From three independent transformations it became clear that U1-70K localises into speckles in 57.5% of analysed cells, whereas U1A and U1C showed speckled pattern only in 13 and 7% of analysed cells, respectively (Figure 4A). Quantification of cells with nucleolar and CB localisation revealed further differences between U1-70K and U1A/U1C proteins (Figure 4B). U1-70K localised in CBs and in nucleoli in 42.5 and 16% of cells, respectively, whereas these numbers for U1A and U1C were 87 and 95% for CBs, and 67 and 60% for nucleoli. The total number of cells showing both nucleolar and CB localisation in combination with either speckled or diffuse nucleoplasmic staining was also dramatically different, being only 6.5% for U1-70K and 66 and 58% for U1A and U1C, respectively (Figure 4B). Percentages of cells showing diffuse nucleoplasmic and CB localisation were similar for all three proteins: 28.5% for U1-70K, 21% for U1A, and 37% for U1C (Figure 4B). A low percentage (7.5%) of cells expressing U1-70K also showed a speckled pattern in combination with CB localisation (Figure 4B). Finally, U1A and U1C were found in speckles in combination with CB localisation (see Figure 3C, U1C-GFP) with frequencies less then 1%, whereby the intensity of speckle fluorescence was significantly lower compared to that of U1-70K.

Taken together, these results suggest that CBs and nucleoli are involved in U1 snRNP maturation. Furthermore, it is obvious that higher proportion of U1-70K accumulates in speckles compared to CBs and nucleoli, whereas U1A and U1C are most abundant in these two nuclear compartments and are rarely found in speckles.

### Co-localisation studies with FP-tagged U1 snRNP proteins

Having established that transiently expressed U1 snRNP-specific proteins assemble correctly into mature snRNP, we asked next whether they co-localise when co-expressed in *Arabidopsis* protoplasts. This was particularly interesting as we observed that U1-70K and U1A/U1C proteins showed strikingly different nuclear localisation patterns.

**Figure 5:**
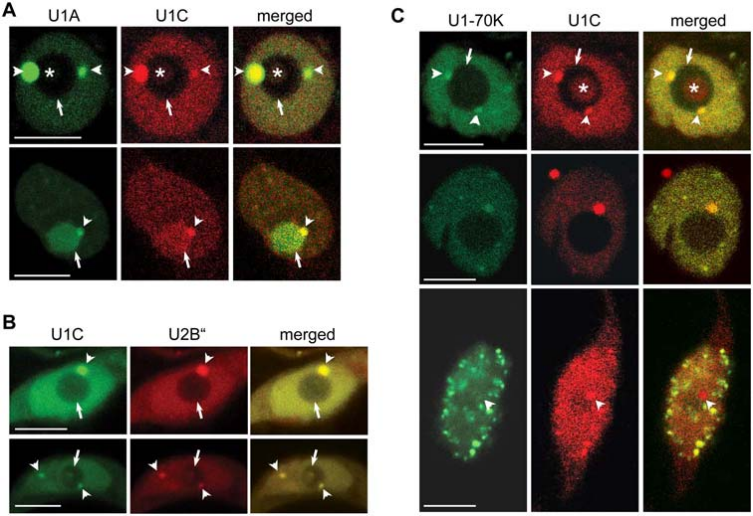
Co-localisation studies with U1 snRNP-specific proteins. (A) Co-localisation of U1A-GFP and U1C-mRFP. (B) Co-localisation of U1C-GFP and U2B″-mRFP. (C) Co-localisation of U170K-GFP and U1C-mRFP. Arrows, arrowheads, and asterisks point to nucleoli, CBs, and nucleolar cavities, respectively. All images are single confocal sections. Scale bars, 8 µm.

As U1A and U1C showed the same frequencies for all nuclear localisation patterns observed we first asked whether they co-localise in *Arabidopsis* protoplasts. Cells co-expressing U1C-mRFP and U1A-GFP showed perfect co-localisation of proteins in CBs, nucleoli, and nucleoplasm (Figure 5A). In addition, co-expression of U1A-GFP and U1C-GFP with U2B″-mRFP (an established marker for CBs in plant cells) [8], [33], [34] resulted in co-localisation of the two protein pairs in CBs and nucleoplasm (Figure 5B). This is in agreement with the fact that at the steady-state in both, plant and animal cells, U2 snRNP accumulates in CBs [8], [33]–[36] and further support our observation that CBs are sites of U1 snRNP biogenesis.

U1-70K was shown to localise primarily in a speckled pattern in the nucleoplasm, although localisation in CBs and nucleoli was also observed (Figures 3 and 4). This was quite different from the localisation pattern frequencies observed with U1A and U1C proteins. Co-expression of GFP-tagged U1-70K with U1C-mRFP resulted in co-localisation of the two proteins in the diffuse nucleoplasmic pool. Co-localisation was also observed in CBs, but only in cells where U1-70K did not show a speckled pattern (Figure 5C). However, fluorescence intensities of U1-70K in CBs were much lower compared to that of U1C (Figure 5C, upper and middle row). Consistent with the fact that U1-70K did not strongly accumulate in nucleoli, very little co-localisation between these two proteins was observed in this compartment (Figure 5C, upper row). Finally, in cells showing a speckled localisation pattern for U1-70K, co-localisation with U1C was not observed in CBs, and very little in speckles (Figure 5C, lower row). This is consistent with the frequencies of different patterns occurring in cells expressing U1-70K and U1C alone (Figures 3 and 4). Also, this indicated that co-expression of two proteins does not influence localisation of each other.

Taken together, our localisation and co-localisation data indicate that the assembly of U1-70K and U1A/U1C proteins into mature U1 snRNP are either following different pathways or different kinetics. U1-70K protein might largely localise directly to speckles, without passage through CBs and nucleoli, which may indicate an additional role for this protein apart from being a U1 snRNP component involved in pre-mRNA splicing.

## Discussion

U1 snRNP is the most simple among the five spliceosomal snRNPs, containing only three snRNP-specific proteins, U1-70K, U1A, and U1C. Previous work on human and animal U1 and U2 snRNPs suggested that snRNP-specific proteins enter the nucleus independent of their cognate RNAs [2], [22], [23], [25], [35], [36]. We could show here, by using biochemical and cell biological approaches that transiently expressed *Arabidopsis* U1 snRNP-specific proteins localise to the nucleus and efficiently assemble into mature snRNPs. If the transport of *Arabidopsis* U1 snRNP-specific proteins into the nucleus follows the same route then we should observe nuclear accumulation of all three proteins with similar kinetics. However, they displayed differential localisations. U1-70K localised exclusively to the nucleus at all time points after transformation whereas U1A and U1C were found in the nucleus and in the cytoplasm even 48 hours after transformation. This difference cannot be accounted for by the different expression levels or the size of the three fusion proteins. First, all three proteins are expressed at similar levels irrespective of the tag used. Second, U1-70K fused to HA tag has a similar size (56 kDa) as GFP-tagged U1A and U1C proteins making it unlikely that the cytoplasmic localisation of U1A and U1C proteins is due to free diffusion of snRNP-free fraction of proteins. It is also possible that GFP or mRFP tags impair nuclear import to some extent. However, nuclear accumulation of the U1-70K (this work) and of all *Arabidopsis* SR proteins analysed [34] was found to be very fast, as no cytoplasmic fluorescence was observed at any post-transformation time point. This indicates that overexpression of the fusion proteins may not be a general limiting factor for efficient nuclear import.

Still, it is not clear why in *Arabidopsis* protoplasts U1A and U1C proteins show cytoplasmic localisation. Animal U1A protein was proposed to accumulate in the nucleus by an active, U1 snRNA independent mechanism [25], [39] whereas U1C protein accumulates in the nucleus by diffusion and nuclear retention [45]. However, neither of these two mechanisms could explain the behaviour of both U1A and U1C *Arabidopsis* proteins. It has been proposed previously that at least some animal U1 and U2 snRNP-specific proteins enter the nucleus independently of U1/U2 snRNAs and that the efficiency of the nuclear import depends on the availability of free U1/U2 snRNAs in the nucleus [22], [23]. We showed previously that the cells expressing GFP or mRFP-tagged U2 snRNP-specific proteins U2A′ and U2B″, in addition to a predominant nuclear localisation, also show cytoplasmic staining [34]. In addition, transient expression of *Arabidopsis* SF3b49 and p14, subunits of the U2 snRNP SF3b subcomplex, as well as a core Sm protein, SmB also resulted in cytoplasmic localisation (our unpublished data). Based on our data and on above reports it is therefore most likely that under overexpression conditions, the available binding sites for U1A and U1C proteins became limited, which obviously leads to cytoplasmic retention of proteins. An additional significant difference between the U1-70K and the U1A and U1C proteins was found. U1-70K was found predominantly in the nucleus in splicing speckles whereas U1A and U1C showed mostly diffuse nucleoplasmic staining. Interestingly, transiently expressed *Arabidopsis* U11-35K protein, a component of the U11 snRNP which is involved in splicing of minor introns [46], was also found only in the nucleus in a speckled pattern [43]. Rapid and predominant speckled localisation of U1-70K and U11-35K may indicate that they localise into speckles without prior association with snRNP. A possible explanation could be the interaction of U1-70K and U11-35K with other speckle components, like for example SR proteins, which are known to interact and co-localise with U1-70K [34], [47], [48] and U11-35K [43] in plant cells. In contrast, U1A and U1C proteins which have not been found to interact with SR or other proteins accumulating in speckles show a rather diffuse nucleoplasmic localisation. Interestingly, the yeast U1C protein was found to bind to the 5′ splice site in the absence of pre-mRNA-U1 snRNP base pairing [49]. In human cells the U1A protein, aside from its role in snRNP function, exists in a snRNP-free fraction which is involved in regulation of its own expression level and in regulation of polyadenylation of various cellular pre-mRNAs [50], [51 and references therein]. Hence, it is well possible that the predominant diffuse nucleoplasmic localisation of *Arabidopsis* U1A and U1C proteins also reflects their additional functions, apart from U1 snRNP and pre-mRNA splicing.

SnRNP biogenesis is a stepwise process, which includes a cytoplasmic and a nuclear phase. However, it is not known how and at which stage of snRNP biogenesis these proteins are incorporated into mature snRNP. By using transient expression of GFP-tagged Sm proteins in mammalian cells it has been shown that, after re-import from the cytoplasm into the nucleus, snRNPs first appear in CBs, then in nucleoli, and finally in speckles [26]. These and other recent data suggested that CBs are final places for snRNP biogenesis [2], [35], [36], [38]. However, in plant and animal cells neither U1 snRNA nor U1 snRNP-specific proteins accumulate in CBs [10], [11], [33], [40], [52]. This is in contrast to U2 snRNA and U2 snRNP-specific proteins which were found in CBs at the steady-state and after transient expression in plant and animal cells [8]–[12], [33]–[36]. This is raising the question whether this nuclear compartment is involved in U1 snRNP biogenesis like in the case of the other four spliceosomal snRNPs. Here, we could clearly show that all three U1 snRNP-specific proteins, when overexpressed in *Arabidopsis* protoplasts, do accumulate in CBs, indicating that CBs are involved in the U1 snRNP biogenesis.

Why would overexpression lead to accumulation of U1 snRNP-specific proteins in CBs? The most plausible explanation would be that overexpression saturates the assembly system, which leads to visualisation of rather fast steps in U1 snRNP assembly. In contrast, under normal expression levels of U1 snRNA and U1 snRNP-specific proteins they might not be detected in CBs simply because they pass vary fast through this nuclear compartment. In that respect, it is also interesting to note that U1 snRNA is not as highly modified as U2 snRNA. Modifications of U snRNAs by scaRNA guided process take place in CBs [27]–[30] and they are necessary for snRNP assembly [31], [32], [53]. Thus, assembling U1 snRNPs most probably do not spend the same time in CBs as U2 snRNP, which contains at least 12 snRNP-specific proteins and the U2 snRNA which is modified on at least 23 places [32].

Our results also clearly show that, in addition to CBs and nucleoplasm, U1 snRNP-specific proteins localise to the nucleoli as well. As already discussed, transient expression of Sm proteins in mammalian cells led to the passage through nucleoli [26], [54]. In addition, internal modifications of U2 snRNA seem to occur in nucleoli of *Xenopus* oocytes [53]. Together these data suggested that the nucleolus might be involved in snRNP biogenesis, although transiently expressed U2 snRNP-specific proteins were not detected in nucleoli of mammalian cells [35]. However, our previous studies with the U2 snRNP-specific proteins, U2B″ and U2A′ [34], and our unpublished data for the U2 snRNP-specific proteins SF3b49 and p14, showed that these proteins also localise to nucleoli. Similarly, we also observed nucleolar localisation of SmB-GFP protein transiently expressed in *Arabidopsis* protoplasts. We could show previously that SR proteins did not localise to CBs and nucleoli upon transient overexpression in protoplasts [34], [55]. Therefore, we conclude that the localisation of U1 snRNP proteins in these two compartments is specific and most likely reflects maturation pathway of U1 snRNP in vivo. Interestingly, a proteomic analysis of the *Arabidopsis* nucleolus revealed that many proteins involved in pre-mRNA splicing, including some SR proteins, some snRNP proteins (i.e. SF3b49, U2A′, SART3, SmD1-D3, G, F), as well as exon-junction complex proteins, which are involved in mRNA export and nonsense mediated decay, localise to some extent to this nuclear compartment [56]. These results together with our data presented here indicate that the plant nucleolus might be actively involved in assembly and/or re-cycling of spliceosomal complexes.

## Materials and Methods

### Plasmids

Plasmids expressing U1 70K-GFP, U1-70K-HA, U2B″-mRFP and SRp34-GFP have been described [34]. Plasmids expressing U1A-GFP, U1A-HA, U1C-HA U11-35K-GFP and U11-35K-RFP have been described [43]. To generate plant expression plasmids encoding GFP and mRFP tagged U1C protein, the coding region of U1C was amplified by using oligonucleotides: U1C 5′ primer GATCG**GTCGAC** *AATAAACC*ATGCCGAGGTATTACTGTG and 3′ primer AGCAT**GGATCC**TTTCTTGGCATACGTGATG, which introduce a *Sal*I (bold) site and a plant translation consensus sequence (italics) in front of the ATG codon and a *Bam*HI site in place of the stop codon, respectively. The PCR products were cut with *Sal*I and *Bam*HI and ligated into the plant expression vectors pDEDH-GFP and pDEDH-mRFP [34], resulting in pU1C-GFP and pU1C-mRFP, respectively.

### Preparation and transient transformation of *Arabidopsis* protoplasts

*Arabidopsis* cell suspension protoplasts were isolated and transformed as described [34]. Transformed protoplasts were collected twenty four hours after transformation and stored at −80°C or were analysed by a laser scanning confocal microscope (Leica).

### Preparation of whole cell extracts from protoplasts, immunoprecipitation, and cellular fractionation

Protoplasts were collected by centrifugation twenty four hours after transformation (15 min, 70×g), frozen in liquid nitrogen, and resuspended in protoplast extraction buffer (PEB400; 50 mM HEPES-KOH pH 7.9, 400 mM KCl, 2.5 mM MgCl_2_, 1 mM EDTA, 1 mM DTT, 0.1% Triton X-100), supplemented with EDTA-free protease inhibitor cocktail (Roche), and further processed as described in Lorkovic et al. [34], [47]. After 15 min centrifugation in an Eppendorf centrifuge at maximum speed at 4°C, the supernatant was mixed with PEB without KCl to adjust KCl concentration to 250 mM (PEB250). Immunoprecipitations and ^32^P-[pCp] labelling of snRNAs were performed as described in Lorkovic et al. [34], [47]. Cellular fractionation of transformed protoplasts into nuclear and cytoplasmic fractions was performed as described by Lambermon et al. [57].

### Confocal microscopy

Images were obtained with a TCS-SP confocal microscope (Leica Microsystems, Heidelberg). GFP and RFP were excited with an ArKr laser at 476 and 568 nm, respectively. GFP was detected at 510 nm to 550 nm, and RFP and mRFP were detected at 630 nm to 680 nm. Images were exported to Adobe Photoshop software and prepared for presentation.

### SDS-PAGE and Western blotting

12% SDS-PAGE was done according to standard procedure. Proteins were transferred onto PVDF membrane (Millipore) and Western blotting was performed according to standard procedure. Rat anti-HA (3F10, Roche) and mouse anti-GFP (Roche) monoclonal antibodies were used at 1∶1,000 dilution. Mouse anti-RBP45 [58] monoclonal and rabbit anti-cFBP (Agrisera) polyclonal antibodies were used at 1∶200 and 1∶5,000 dilutions, respectively. Secondary antibodies, goat anti-rat (Sigma), goat anti-mouse (Biorad) IgGs, and goat-anti rabbit (Biorad) conjugated with horseradish peroxidase were used at 1∶10,000 dilutions. The blots were developed using an enhanced chemiluminescence kit (AmershamPharmacia Biotech).
